# A delayed diagnosis of Pallister-Hall syndrome in an adult male following the incidental detection of a hypothalamic hamartoma

**DOI:** 10.1038/s41439-018-0031-9

**Published:** 2018-11-12

**Authors:** Eliza Courtney, Du Soon Swee, Diana Ishak, Joanne Ngeow

**Affiliations:** 1 Cancer Genetics Service, Division of Medical Oncology, National Cancer Centre Singapore, Singapore, 169610 Singapore; 2 Department of Endocrinology, Singapore General Hospital, Singapore, 169608 Singapore; 3 Lee Kong Chian School of Medicine, Nanyang Technological University, Singapore, 308232 Singapore; 4 Oncology Academic Clinical Program, Duke-NUS Medical School, Singapore, 169857 Singapore; 5 Institute of Molecular and Cell Biology, Agency for Science Technology and Research (A*Star), Singapore, 138673 Singapore

## Abstract

Pallister-Hall syndrome is a rare autosomal dominant condition that is associated with polydactyly and hypothalamic hamartoma and is caused predominantly by frameshift or nonsense pathogenic variants in the *GLI3* gene. The majority of cases are identified during childhood; however, rare reports of diagnoses during adulthood exist. Here, we describe the identification of a novel nonsense *GLI3* pathogenic variant in an adult male following the incidental detection of a hypothalamic hamartoma.

Pallister-Hall syndrome (PHS; MIM# 146510) is a rare autosomal dominant condition first described in 1980 as a lethal condition in the neonatal period^1,2^, but has since been identified to demonstrate wide phenotypic variation as new cases emerged. A clinical diagnosis of PHS is typically made in the presence of mesoaxial polydactyly and hypothalamic hamartoma^3,4^, although others have suggested the criteria should be modified to include postaxial polydactyly^5^. Other features associated with PHS includes: bifid epiglottis; anal, renal, genitourinary, and pulmonary abnormalities; nonpolydactyly skeletal anomalies; and developmental delay.

Once there is a clinical suspicion of PHS, the diagnosis can be confirmed by molecular testing of the *GLI3* zinc finger transcription factor gene on 7p14.1^6^. Pathogenic variants in the *GLI3* gene are responsible for several conditions in addition to PHS, including Greig cephalopolysyndactyly syndrome (GCPS; MIM# 175700), acrocallosal syndrome (MIM# 200990), and nonsyndromic polydactyly (MIM# 174700). The GLI3 protein is a modulator of the *Sonic hedgehog* (SHH) pathway and can act as either a transcriptional activator or repressor, depending on the presence of SHH^7^. Robust genotype–phenotype correlations have been demonstrated for both PHS and GCPS^3,8^—PHS is usually caused by truncating variants in the middle third (between nucleotides 1998 to 3481) of *GLI3*.

The exact prevalence of PHS is unknown, and to date, more than 100 cases have been described in the literature^3,4,8–10^. It is hypothesized that there may be many PHS cases that remain unidentified, either due to misdiagnosis with another polydactyly condition or because of a lack of awareness. Early intervention for many of the manifestations, particularly endocrine and developmental, is important. Here, we describe a case of PHS identified in adulthood to highlight missed opportunities that can delay diagnosis.

A 21-year-old Chinese male who was undergoing magnetic resonance imaging (MRI) of the internal auditory meatus (IAM) for investigation of asymmetrical mild-moderate low frequency sensorineural hearing loss diagnosed since childhood was incidentally found to have a large sellar and suprasellar mass. Subsequent MRI of the pituitary fossa indicated it to be likely a hypothalamic hamartoma. He was seen by an endocrinologist for further evaluation of his pituitary hormones. Clinically, the patient was euthyroid, and there was no postural hypotension elicited. Testicular size was normal at 20 ml bilaterally, and secondary sexual characteristics were present. On biochemical evaluation, other than a borderline peak serum cortisol response of 490 nmol/l following a short Synacthen test (normal response: ≥500 nmol/l), the rest of the pituitary function was well intact. Based on a suspicion of PHS, the patient was referred for a genetics assessment.

Detailed clinical (Table 1) and family (Fig. 1a) histories were taken. The patient was born to nonconsanguineous parents following an uneventful pregnancy with normal spontaneous vaginal delivery at full-term. His mother reported no teratogen exposure during pregnancy. At birth, he was hypotonic and weighed 3800 g (81st centile). He presented with bilateral polydactyly: left hand mesoaxial and postaxial polydactyly (3rd–4th metacarpal syndactyly and bifurcation of 5th metacarpus) and right hand mesoaxial and postaxial polydactyly (3rd–4th metacarpal syndactyly and trifurcation of 5th metacarpus). He underwent several corrective surgeries before 2 years of age. At 6 months, he was noted to have episodes of limb jerking for a period of 2 weeks and underwent an electroencephalogram (EEG) and pediatrician review, which detected no abnormality (no further recurrence was noted). Delayed speech and motor development was noted at age 2 years, and he attended speech, language and occupational therapy at a tertiary children’s hospital. His milestones were delayed: he walked at 24 months; he acquired day- and night-time bladder control at 30 and 45 months, respectively; he acquired bowel control at 30 months; he used single words at 30 months, and he used his first phrases at 42 months. At age 11.5 years, he was assessed to have good cognitive ability but displayed difficulties with social interaction, communication, emotional regulation and motor skills (using WISC-IV, ADI-R, and ADOS). He had precocious puberty at age 8 years and ceased growing at approximately age 13 years. His current height is 163.0 cm (significantly shorter than his sibling), and his weight is 52.7 kg. He has a history of prognathism and underwent jaw realignment surgery at age 14 years. The patient reported recent episodes of intense anxiety accompanied by an inability to move his upper limbs and shaking of lower limbs, which lasted approximately 15–30 min. Subsequent EEG and neurology assessments reported no abnormalities. He is asymptomatic for bifid epiglottis; renal ultrasound was normal; and anal and genitourinary anomalies were absent in this patient. His occipital frontal circumference is 56.0 cm, and he has low-set, prominent ears.

**Table 1:** A comparison of the clinical manifestations identified in our patient, with two published PHS case series

Clinical manifestation	Proband	Johnston et al.^8^ ^a,b^	Démurger et al.^9^ ^a,b^
Mesoaxial polydactyly	+	48% (10/21)	97% (29/30)
Postaxial polydactyly	+	48% (10/21)	40% (12/30)
Hypothalamic hamartoma	+	100% (12/12)	97% (29/30)
Bifid epiglottis	NA	44% (4/9)	100% (19/19)
Developmental delay	+	21% (3/14)	39% (9/23)
Seizures	+	13% (2/15)	46% (11/24)
Anal anomalies	−	48% (10/21)	Imperforate anus, anal/vaginal fistula
Renal anomalies	−	41% (7/17)	Loss of kidney function, kidney hypoplasia, vesicoureteral reflux
Genital anomalies	−	48% (10/21)	Genital hypoplasia, vaginal atresia, urogenital sinus, hydrometrocolpos, anal/vaginal fistula
Pulmonary anomalies	−	50% (4/8)	–
Nail hypoplasia	NA	69% (9/13)	86% (21/24)
Endocrine manifestations	Precocious puberty	GH deficiency, panhypopituitarism	Precocious puberty, GH deficiency, hypothyroidism, cortisol/thyroid deficiency
Limb anomalies	Y-shaped metacarpal, syndactyly, brachydactyly, short stature	Y-shaped metacarpal, syndactyly, brachydactyly, oligodactyly, mesomelia, overlapping toes, athrogryposis	Feet polydactyly, hypoplastic iliac bone, short limbs, syndactyly
Craniofacial/dental anomalies	Prognathism, pointed teeth	Microagnathia, retroagnathia, agnathia, choanal atresia, cleft palate	Dental hypoplasia (47%; 9/19), dental crowding, oral frenulum, laryngeal cleft
Other	Bilateral hearing loss, hypotonia, difficulties with social interaction, communication, emotional regulation, motor skills	Cardiac anomalies, IUGR, adrenal hypoplasia	Uni-lateral and bilateral hearing loss, hypotonia, tracheal stenosis

(+) Reported in individual(s), (−), Absent from individual(s); *GH* growth hormone, *IUGR* intrauterine growth restriction, *NA* not assessed or data not available

^a^Prevalence of manifestation in individuals with PHS according to two case series provided where available. Prevalence is determined by the number of individuals with the manifestation as a proportion of the total number of individuals assessed for the manifestation

^b^Manifestation descriptions provided when prevalence (%) was not reported

**Fig. 1:**
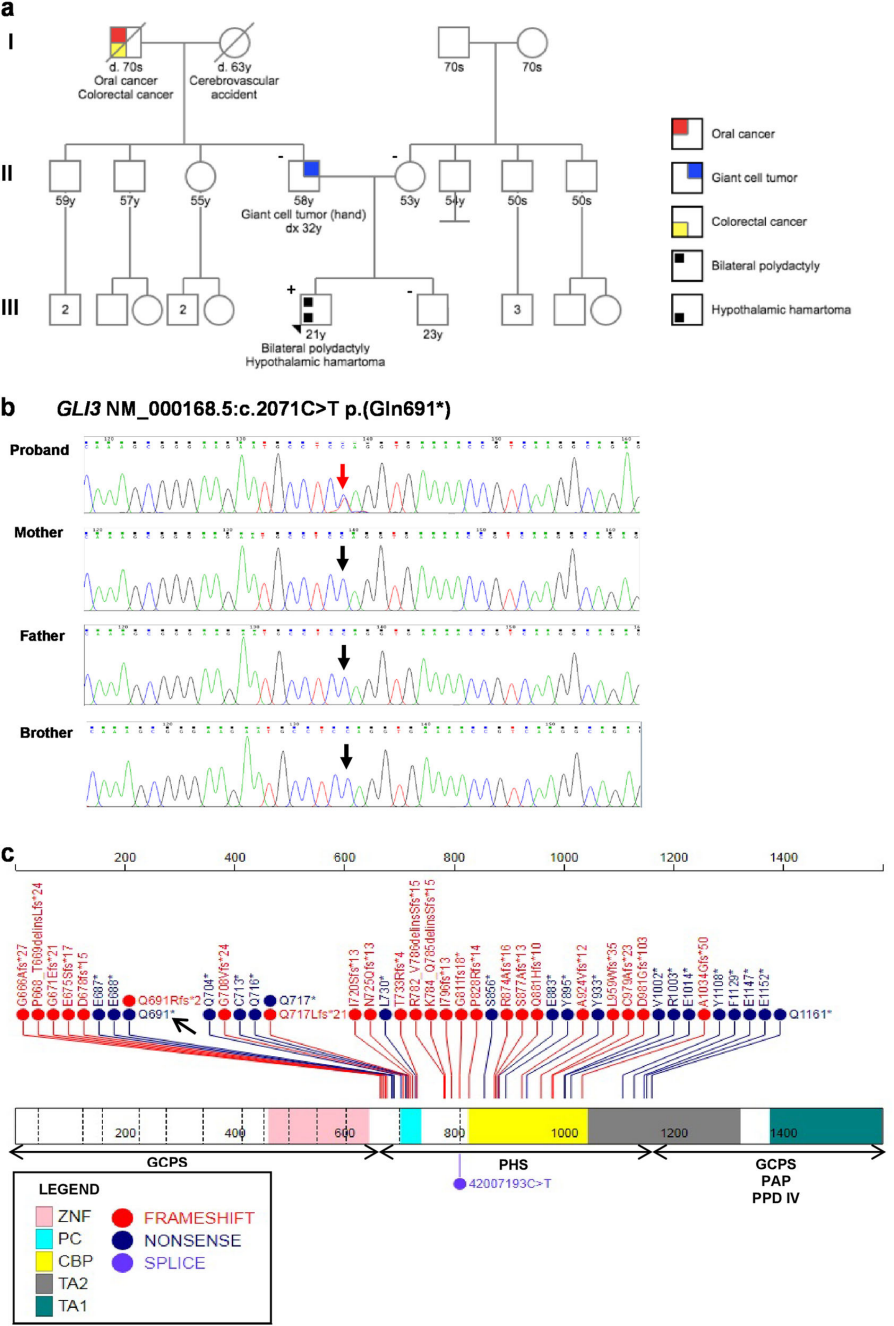
**a** Pedigree displaying the family history (Progeny LLC Free Online Pedigree Tool Application, accessed 14 April 2018). The proband is indicated by the black arrow. (+), indicates the *GLI3* variant was detected in peripheral blood DNA; (-), indicates the *GLI3* variant was not detected in peripheral blood DNA. Both maternal and paternal families are Chinese. **b** Partial sequence chromatograms of *GLI3* in the proband and his first-degree relatives. The red arrow indicates the presence of *GLI3* NM_000168.5:c.2071C>T p.(Gln691*), and the black arrows indicate the wild-type allele. According to the 2015 American College of Medical Genetics and Genomics (ACMG) guidelines for variant interpretation^14^, the *GLI3* nonsense variant is considered pathogenic. **c** Schematic view of the *GLI3* gene structure and the pathogenic variants associated with Pallister-Hall syndrome (PHS) reported to date (St. Jude PeCan ProteinPaint application, accessed 14 April 2018). Black arrow indicates the novel nonsense variant detected in our patient. ZFD, zinc-finger domain (amino acids, aa, 462–645); PC, proteolytic cleavage site (aa 703–740); CBP, cyclic AMP-binding protein-binding domain (aa 827–1132); TA2, transactivation domain (aa 1044–1322); TA1 transactivation domain (aa 1376– 1580). The majority of PHS-causing pathogenic variants are frameshift and nonsense and affect the middle third of the *GLI3* gene (aa 666–1161). Pathogenic variants in the remaining sections of *GLI3* cause Greig cephalopolysyndactyly syndrome (GCPS), postaxial polydactyly A/B (PAP), and preaxial polydactyly type IV (PPD IV)

He has no family history of PHS or PHS-associated manifestations (Fig. 1a). His father was diagnosed with a giant cell tumor of his left distal radius at age 33 years (verified by medical report).

The patient’s peripheral blood DNA was analyzed using the Syndromic Macrocephaly/Overgrowth Panel by the GeneDx, Inc. laboratory^11^, which included 29 genes (*AKT3, BRWD3, CCND2, CHD8, CUL4B, DNMT3A, EZH2, GLI3, GPC3, HEPACAM, HERC1, MED12, MTOR, NFIA, NFIX, NSD1, OFD1, PHF6, PIK3CA, PIK3R2, PPP2R5D, PTCH1, PTEN, RAB39B, RNF135, SETD2, SNX14, TBC1D7, UPF3B*). Although *GLI3* testing alone would have been sufficient in this case, many of the phenotypes associated with these genes have significant overlap. The complete coding regions and splice site junctions of the included genes were enriched and sequenced with paired-end reads on an Illumina platform. Copy number variant (CNV) calling was included. The reads were assembled and aligned to reference sequences (NCBI RefSeq transcripts and human genome build GRCh37/UCSC hg19). A novel heterozygous nonsense pathogenic variant in *GLI3*, NM_000168.5:c.2071C>T p.(Gln691*), was detected (Fig. 1b). In-house Sanger sequencing of peripheral blood DNA from his parents and brother did not detect the *GLI3* variant; therefore, his mutation is likely to be de novo (Fig. 1b), although germline mosaicism cannot be excluded and has been previously reported in PHS^12^. Additionally, he was found to be hemizygous for a variant of uncertain significance (VUS) in *MED12*, NM_005120.2:c.1031C>A p.(Thr344Asn), located on the X chromosome. His mother was heterozygous and brother hemizygous for the *MED12* VUS, and his father was excluded as a carrier. His brother had no *MED12*-associated manifestations and had normal intelligence, suggesting the variant is less likely to adversely affect *MED12* function. Autosomal dominant inheritance and reproductive options, including prenatal diagnosis and preimplantation genetic diagnosis, were discussed with the patient. He will continue to follow-up regularly with endocrinology and neurology. Written research consent was obtained from all four family members.

There have been more than 40 *GLI3* pathogenic variants reported to be associated with either PHS or sub-PHS, where a diagnosis is suspected but does not meet the diagnostic criteria (Fig. 1c). While the majority are frameshift and nonsense mutations, one splicing variant has been reported^8^. Approximately a quarter of all known PHS cases have been caused by a de novo pathogenic variant^4^. The *GLI3* variant detected in our patient falls between the zinc-finger domain and the proteolytic cleavage site. It is located within the middle third of the *GLI3* gene where all PHS-causing pathogenic variants have been identified, providing further support for the well-established genotype–phenotype correlation.

In this report, we describe a case of PHS caused by a novel *GLI3* nonsense variant that was not diagnosed until adulthood, despite multiple points of contact with pediatric and developmental health services throughout the patient’s childhood. Although the patient presented with several features at birth and during early childhood that would warrant a genetics assessment, he was not referred. Only a handful of PHS cases diagnosed during adulthood have been reported^5,13^. The majority are born with polydactyly, but a diagnosis of PHS is only suspected following a subsequent event, such as the presence of epileptic seizures, precocious puberty, hypothalamic hamartoma, or the birth of offspring with a more severe phenotype. The diagnosis of precocious puberty in our patient, in combination with bilateral polydactyly, should have raised the suspicion of PHS. More than a decade passed before a diagnosis of PHS was made, following the incidental finding of the hypothalamic hamartoma. This case adds to the literature of PHS diagnosed in adulthood, which together serve to bring attention to the missed opportunities for genetic testing and subsequent diagnosis. Earlier diagnosis has the potential to alleviate the burden on patients and their families when there is a lack of a definitive reason for their medical history. This is especially important for PHS manifestations, where early detection and management can help improve outcomes, such as endocrine and developmental manifestations. Further education is required to raise awareness among health professionals who may encounter individuals with PHS at early stages of life, such as surgeons and pediatricians.

## Data Availability

The relevant data from this Data Report are hosted at the Human Genome Variation Database at 10.6084/m9.figshare.hgv.2402.
